# Giving permission to care for people with dementia in residential homes: learning from a realist synthesis of hearing-related communication

**DOI:** 10.1186/s12916-019-1286-9

**Published:** 2019-03-04

**Authors:** Brian Crosbie, Melanie Ferguson, Geoff Wong, Dawn-Marie Walker, Stevie Vanhegan, Tom Dening

**Affiliations:** 1 University of Nottingham, Nottingham, UK; 2 National Institute for Health Research (NIHR) Nottingham Biomedical Research Centre, Ropewalk House, 113 The Ropewalk, Nottingham, NG1 5DU UK; 3 Hearing Sciences, Division of Clinical Neuroscience, School of Medicine, University of Nottingham, University Park Campus, Nottingham, NG7 2RD UK; 4 Queens Medical Centre, Nottingham University Hospitals NHS Trust, Derby Road, Nottingham, NG7 2UH UK; 5 University of Oxford, Oxford, UK; 6 University of Southampton, Southampton, UK; 7 Patient and Public Involvement, Nottingham, UK

**Keywords:** Dementia, Hearing loss, Hearing communication, Care home, Permission, Realist synthesis

## Abstract

**Background:**

Managing hearing communication for residents living with hearing loss and dementia in long-term care settings is challenging. This paper explores how care can be effective in optimising hearing communication for residents living with dementia. We argue that the underlying notion of permission or authorisation allows care staff to do what they know will be effective in providing person-centred care that enhances hearing communication. The paper also indicates that this notion of permission can usefully be applied to other areas of care home practice.

**Methods:**

To address hearing-related communication in care homes, we conducted a realist synthesis (RS). As a theory-driven approach to reviewing literature, it also uses expert opinion to understand complex health situations. Using RS, we developed a theory surrounding the management of hearing-related communication in care homes. Applying formal processes to the literature search and data extraction, the analysis uncovered relevant mechanisms and contexts to help confirm, refute or refine our understanding of how hearing communication could be improved.

**Results:**

Forty-three papers were selected for the realist synthesis. The documents were analysed to construct five context-mechanism-outcome configurations (CMOCs). The CMOCs represent possible care interventions to optimise hearing-related communication in care homes for person living with dementia and hearing loss (PLWDHL). They include leadership promoting positive regard and empathy through person-centred care, communication training for staff, ‘knowing the person’ and relationship building for responsive awareness of residents’ hearing needs, maintaining and monitoring hearing communication through care planning, and managing noise in the care home environment.

**Conclusions:**

Leadership that provides appropriate training and resources is likely to enhance knowledge and skills, leading to staff feeling able and equipped to respond to the hearing-related communication needs of PLWDHL. Collaboration with local hearing services is likely to raise awareness of hearing loss among care home staff. Importantly, care staff require a sense of permission from leadership, to work with knowledge and autonomy in the interest of residents living with dementia and hearing loss.

**Electronic supplementary material:**

The online version of this article (10.1186/s12916-019-1286-9) contains supplementary material, which is available to authorized users.

## Background

This paper explores how effective care is provided for residents in long-term care settings (variously termed nursing homes, residential care or, as in this paper, care homes). The argument presented is that the underlying notion of permission or authorisation of care staff to do what they know will be effective applies to any feature of personal care that is provided. However, the approach taken here is to examine one specific area, by way of example, to illustrate this concept. The study used as the illustrative example is a realist synthesis of the hearing-related communication needs of care home residents living with dementia.

### Dementia and hearing loss

Hearing loss is related to an increased incidence of dementia [1]. People with hearing loss have accelerated brain atrophy [2], and not wearing hearing aids is suggested to be associated with accelerated cognitive decline [3]. In addition, dementia causes a range of language and sensory processing impairments, including central auditory dysfunction [4]. The relationship between hearing loss and both dementia and cognitive decline is seen as a research priority by NICE [5] and the James Lind Alliance [6]. In addition, a recent Lancet report [7] identified hearing loss in midlife as the top potential modifiable risk factor for later life onset of dementia.

Both dementia and hearing loss are common problems among older people in residential care settings. Each can contribute to poor communication between the resident and other people. Management of hearing-related communication problems in care homes is variable and usually based on limited evidence.

The prevalence of hearing loss in care home residents aged 65+ is estimated between 70% [8] and 90% [9, 10], and the prevalence of dementia is around 75% [11]. Residents in care homes are disproportionately likely to be affected by hearing loss [12]. Hearing loss leads to significant difficulties with interpersonal communication, and the consequences of this are far-reaching, and can lead to social withdrawal and isolation, stigma, depression [13–15], and reduced quality of life [16]. Impaired hearing-related communication also contributes to problems with behaviour such as aggression [17]. Furthermore, the combination of dementia with hearing loss may be compounded by factors within the care home, such as high levels of background noise [18], and when the resident or staff member do not speak the same first language, furthering the impact on effects such as social isolation, withdrawal, and depression (e.g. Hyer et al. 2005; [19]).

Similarities in the behaviours and symptoms of untreated hearing loss and dementia (e.g. social isolation, repeatedly asking questions, stereotyped/inappropriate word use, difficulty following conversation) can lead to misdiagnosis or false identification of dementia [20]. It has been suggested that hearing loss can masquerade as dementia [21]. People diagnosed with dementia have a high rate of untreated hearing loss [20], and fitting hearing aids can reduce problematic behaviours in those with dementia [22]. Though findings are variable, [23] reported improvements in global ratings but not in cognition, behaviour or psychiatric symptoms.

Care home residents with dementia are more likely to have hearing loss than are residents in the community [24], yet hearing aid use is poor [25]. Barriers to good hearing healthcare in care homes include difficulties with residents using hearing aids because they fit poorly, do not work properly, batteries run out, or residents cannot handle them with many (86%) needing help [25]. Staff have difficulty in discriminating the relative contributions of hearing loss and dementia to communication breakdowns with both conditions leading to excessive communication disability and communication vulnerability [26]. The corollary of these barriers to communication is that low priority is placed on managing residents’ hearing needs [27]. Often, the pressure of time and resources requires care home staff to focus on medical needs, deemed to be more pressing than time spent in social interaction.

There is a dearth of research on the dual impact of hearing loss and dementia on communication in care homes, although it is recognised that this is both complex and multifactorial [25]. Furthermore, there are complex interactions of person and environment in the care home setting [28]. Echalier [12] looked at hearing loss in care homes, though not specifically among residents with dementia, and identified three areas for action: (1) intervene earlier in hearing loss, (2) meet communication needs in care homes, and (3) improve hearing aid use and management. This topic area is complex, the outcomes of any interventions are often dependent on the specific context, and there is a paucity of research data on dementia and hearing loss occurring together to inform decision-making. What is perhaps clear is that much of the onus on improving the hearing-related communication for individuals with dementia lies with front-line staff. However, staff often lack awareness of hearing problems and its consequences, receive poor training, and have poor knowledge of hearing aids and communication strategies [29].

### Realist synthesis and aims of this study

Realist synthesis (RS) is a theory-driven approach to reviewing literature from a range of data sources [30], which makes it a suitable methodology for evaluating complex situations, such as dementia and hearing loss in care homes. It interrogates the questions of what works for whom, how, when, and why? We undertook a RS to address the following question:

How, why, to what extent, for whom, and in what circumstances do interventions work to manage important and relevant outcomes for people with hearing loss and dementia in care homes?

RS attempts to understand the underlying generative mechanisms in complex situations [31] that produce outcomes, in this case, that either ensure or prevent the optimal management of hearing-related communication for people living with dementia and hearing loss (PLWDHL). RS seeks to gain an understanding of what works by disclosing the context (C) conditions that trigger causal mechanisms (M) to produce both expected and unintended outcomes (O) [32, 33]. In conducting the review, we were also aware of the possibility that mechanisms identified might be relevant across other areas of care provided in care homes. Clearly, if common mechanisms operate, then this might go some way to predict what would be effective in other types of task, not just hearing-related communication.

The review was registered with PROSPERO (CRD42017074790) [34].

## Methods

This RS, Optimising hearing-Related communication in Care Home Residents with Dementia (ORCHARD), had four stages: programme theory development, searching the literature and eligibility processes, data extraction,, and analysis and synthesis. When undertaking our RS, we followed the RAMESES quality and publication standards [35].

### Programme theory development

The initial phases of the review concentrated on devising an initial programme theory. The programme theory was devised from ORCHARD research team members’ prior knowledge of the field of dementia and hearing-related communication. Our project team comprised context experts in dementia and old age psychiatry (TD), audiology (MF), realist methodology and general practice (GW), research methods (DW, BC) and patient and public involvement (SV). Our insights were augmented through an initial scoping of known research literature held in the personal collections of members of the team, including primary research, intervention literature, and expert opinion [33]. The initial PT encompassed core themes derived from our scope of the literature and expert opinion input. These themes highlighted the existing concerns surrounding dementia and hearing loss in the care home environment.

The next step in the early phase of the study was to present the initial programme theory to the context expert group (CEG, see below for description). The CEG provided feedback on the content and coherence of the programme theory and the emerging themes. This task enabled an early ‘firming up’ of five themes and also gave conceptual clarification to the empirical content of the themes. Using these processes, we developed the five themes into five theory areas which captured the focus of the overall review. We iteratively developed context-mechanism-outcome configurations (CMOCs) for each of these five theory areas. During this review, our initial programme theory and CMOCs were tested and refined through focused CEG discussions and feedback.

### Context expert group

To ensure a ‘real-world’ veracity to the RS, a group of context experts involved with dementia and hearing loss was convened. This context expert group (CEG) was formed of twenty individuals and comprised gerontologists, audiologists, care home staff and management, stakeholder organisations, and individuals who had first-hand experience of caring for family members with dementia and hearing loss, living in a care/nursing home environment.

The CEGs convened on seven occasions during the study with on average nine members variously attending across all the meetings. The purpose of the first two meetings was to present to the initial programme theory and the five embryonic themes for scrutiny. In the meetings, facilitators (DW, SV, BC) sought feedback and consensus on the broad issues present in the programme theory and themes. It was also an opportunity to gain clarification of core concepts, for example, person-centred care and maintenance of assistive hearing devices. Information gathered during the CEGs informed the literature search and data analysis, carried out by the research fellow (BC). In subsequent CEGs, configured CMOCs were presented to the group for further discussion and feedback for refinement of the programme theory. With direct experience of the topic, CEG members, particularly care home staff and carers of family members with dementia and hearing loss, added greatly to the refinement of the context features of each theme and then CMOCs. In effect, the advice and feedback from the CEGs proved invaluable for the iterative testing and refinement of our initial PT and CMOCs. The final CEG was used to scrutinise the recommendations that were informed by the refined PT and CMOCs.

### Literature search

There were three linked search phases. The first phase search used the key terms to build the initial PT and themes. The second phase involved citation tracking of selected studies from the first phase. The third phase involved a search of grey literature. The second and third phases were used to enhance the yield of papers and to find more data to test our initial programme theory and CMOCs. Thus, for example, on the topic of staff training, we needed to focus the search onto communication training in general for care home staff. All searches were developed, piloted, and carried out by our information specialist.

The databases and search terms used in the first phase search are shown in Table 1. Search dates were from 1980 to March 2018. It was necessary to combine three search terms in order to manage the numbers of references otherwise identified. After an initial screening by BC of the title, abstract, and keywords, an inter-rater reliability check was conducted on a random 10% sample of papers by TD and GW. Any disagreements were resolved by discussion. Following the initial screening, the remaining papers were read in full by BC to determine eligible papers for inclusion in the review.

**Table 1:** Search terms and databases

Search terms	
Dementia [OR] Alzheimer’s disease [OR] vascular dementia [OR] dementia with Lewy bodies [OR] ‘cognitive impairment’ [AND] ‘care home’ [OR] ‘residential care home’ [OR] ‘residential home’ [OR] ‘long-term care’ [OR] ‘nursing home’ [AND] ‘deafness’ [OR] ‘hearing loss’ [OR] ‘hearing impairment’ [OR] ‘hearing aids’	
Databases	
Medline, Embase, PsycINFO, Cinahl, ISI Web of Science, British Nursing Index (BNI) Cochrane Library, U.S. National Library of Medicine- Clinical trials.gov	

Inclusion criteria for the review were (1) papers with a focus on dementia and hearing loss in older people living in long-term care, (2) primary research should have been carried out with adequate rigour (as agreed by discussion within the research team) so as to produce sufficiently trustworthy results, and (3) other documents, such as opinion pieces, practice recommendations, and qualitative reviews if the topic area was relevant to the topic area. The quality of grey literature publications lay in the ‘… contribution that each one makes to the developing synthesis’ [30] p. 87. We also made judgements about rigour at the level of the arguments that we used to support our CMOCs and programme theory. Within realist reviews, it is permissible to use data generated from research methods (or not) of ‘low’ or ‘very limited’ quality. Nevertheless, the specific data extracts might provide useful information to build theories based on arguments with good explanatory powers. To judge the explanatory powers of our theories, we used three criteria: consilience, simplicity, and analogy. Put briefly, theories have consilience if they are able to explain as much of the data as possible; simplicity, if they do not have too many ad hoc exceptions; and analogy, if they fit in with the existing knowledge of the topic area. Readers interested in using such an approach may wish to see additional resources [36].

The second phase of the search strategy involved citation tracking of the papers included from the first phase search. The information specialist used the cited reference search function in ISI Web of Science and Scopus databases and also the ‘cited by’ function on Google Scholar. All of the cited references for the key papers were imported into Endnote, and any duplicate records (or references that had already been identified) were removed.

The third phase of the search was to identify grey literature by searching websites for policy papers, publication of practice recommendations, conference proceedings, or opinion and blog pieces, all pertaining to hearing loss and dementia in the care home context.

Throughout the screening and eligibility phase, the papers were considered for their potential to provide data on context, mechanism, and outcome components. We also examined whether they addressed the emerging five theory areas surrounding dementia and hearing loss in residential care. For example, if a study described the delivery of dementia communication training to care home staff, did it offer insights on how the training ‘worked’, and what aspects of the training content might trigger the promotion of best practice around staff communication with residents?

It was not required that a paper provided all three elements of a CMOC. (Indeed, in most cases, papers only contained data that informed part of any CMOC). At each phase of the search, a CEG meeting was convened. The output from those meetings iteratively informed any subsequent search for material.

### Data extraction

The final included papers were read in full. Data relating to the characteristics of the papers, where available, were extracted into an Excel spreadsheet. This included data on authors, methods, study aims, research question(s), study setting, and a summary of salient results. The spreadsheet was used as our database. From the full texts of the selected papers, the researcher (BC) extracted relevant sections of text into the qualitative analysis software NVivo [37] which were then used to build up iterative configurations of our CMO configurations.

### Analysis and synthesis

In the first phase of the analysis, broad sections of extracted data were assigned by content salience to one of the five broad theory area nodes constructed within NVivo. Each theory area had been derived from scoping the literature and the initial programme theory.

The second phase involved analysing the data within the broad theory area nodes, the purpose being to interpret whether data were functioning as context, mechanism, or outcome and if so, which CMOC it belonged in the devised context, mechanism, and outcome sub-nodes in NVivo. These analyses were undertaken by BC and presented, discussed, and refined at regular project team meetings.

To construct our CMOCs, we drew data from all the included papers. Our CMOCs were developed, confirmed, refuted, or refined using data that came from more than one included paper. We also compared and contrasted the reported data across included papers to gain an understanding of the behaviour of possible mechanisms within different contexts. Drawing on data from more than one included paper to construct our CMOCs also enabled us to further our understanding of the connected and complex nature of interactions among the CMOCs contained within our final programme theory. We give a working example of our analysis and CMOC construction in Additional file 1.

To reduce potential bias, BC provided the research team with regular analyses reports including the node structure and illustrative data extracts assigned to them. Along with data extracts, BC provided a precis of the analytical judgement which drove the assignment of data to the CMOCs. This was discussed further during team meetings. Along with the deliberations of the research team, the emerging CMOCs were also scrutinised and further refined by members of the CEG.

## Results

### Search results

**Fig. 1:**
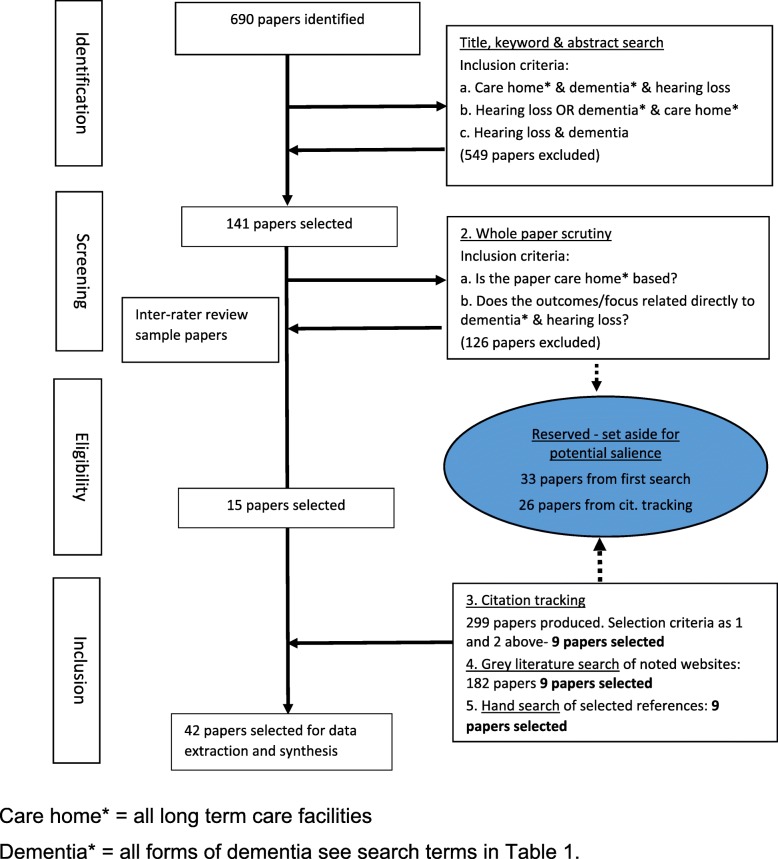
Flow diagram of the literature search. Care home* = all long term care facilities. Dementia* = all forms of dementia see search terms in Table 1

The results of the search process are shown in the flow chart Fig. 1. Altogether, 1171 papers were scrutinised, using the inclusion criteria stated in Fig. 1, for potential inclusion in the review. In all, a final 43 papers were identified for data extraction and synthesis. Fifteen were identified by the first phase search, 9 from citation tracking, 9 from grey literature, and an additional 9 papers were selected during analysis because of their salience in addressing particular areas of the programme theory. There were very few papers specifically concerned with training care staff about hearing-related communication, so for this topic, we added a small number of papers about dementia communication training in general that were identified by hand searching references lists of the core review papers.

Of the papers included, 23 were about dementia and hearing/hearing aid management, 3 about hearing assessment, 12 about staff training and development, 3 were recommendations or guidelines, and 2 were about care.

### Document characteristics

Table 2 shows the characteristics and focus of the papers included in the review.

**Table 2:** Review papers

Study no.	Author	Country	Title	Year	Type (e.g. journal article, book, review)	Focus
[49]	Alzheimer’s Society	UK	Challenges facing primary carers of people with dementia	2012	Published institute report	The report provides recommendation for the long-term management of hearing loss with dementia.
[61]	Allan et al.	UK	Deafness and dementia: what you need to know	2006	Patient and public information document	Advice to professional and layperson on managing dementia and hearing loss.
[64]	Adams-Wendling et al.	USA	Nursing management of hearing impairment in nursing facility residents	2008	Journal article—evidence-based practice guideline	The report provides evidence-based practice guidelines to front-line staff in nursing homes, highlighting the competencies need to communicate effectively with hearing-impaired residents.
[43]	Beer et al.	USA	Communicating with patients who have advanced dementia: training nurse aide students	2012	Journal article—survey-based quantitative study	Findings indicate a need for a person-centred approach to dementia education; limited time was cited as a barrier to education and training; flexible small group training which is practically based.
[39]	Beer et al.	Australia	Current experiences and educational preferences of general practitioners and staff caring for people with dementia living in residential facilities	2009	Journal article—mixed methods study	Findings indicate a need for a person-centred approach to dementia education; limited time was cited as a barrier to education and training; flexible small group training which is practically based.
[52]	Burnip et al.	Australia	Staff perceptions of communication difficulty among nursing home residents	1996	Journal article—questionnaire-based quantitative study	The study investigates nursing home staffs’ perceptions of communication difficulties for residents and hearing and vision impairment.
[29]	Burnip et al.	Australia	Staff knowledge regarding hearing loss and communication among nursing home residents	1997	Journal article—quantitative survey	The study gains an understanding of nursing homes staff perspectives on and response to hearing impairment among residents, with a particular focus on dementia.
[50]	Clare et al.	UK	AwareCare: a pilot randomised controlled trial of an awareness-based staff training intervention to improve quality of life for residents with severe dementia in long-term care settings	2013	Journal article—staff training/randomised trial	Awareness training for care staff on the communication efforts of residents with dementia in long-term care.
[25]	Cohen-Mansfield et al.	USA	Hearing aid use in nursing homes, part 1: prevalence rates of hearing impairment and hearing aid use	2004	Journal article—cross-sectional survey/questionnaire and interviews	Assessing hearing impairment and hearing aid use among residents.
[46]	Cohen-Mansfield et al.	USA	Hearing aid use in nursing homes, part 2: barriers to effective utilization of hearing aids	2004	Journal article—cross sectional survey/questionnaire and interviews	Assessing hearing impairment and hearing aid use among residents.
[56]	Cohen-Mansfield et al.	USA	Hearing aids for nursing home residents: current policy and future needs	2006	Journal article—review	Policy focused review on the management of hearing communication in care homes.
[42]	De Vries	UK	Communicating with older people with dementia	2013	Professional journal article—review and professional development	Recommendations made for the management of communication for people with dementia.
[51]	Eadie-Rutten	Canada	The Hearing Education and Access for Residents (H.E.A.R.) project in a geriatric and chronic care hospital	1992	Journal article—report on communication training programme	Training programme to improve care staff/patient communication in long-term hospital care.
[12]	Echalier	UK	A world of silence: the case for tackling hearing loss in care homes	2012	Published institution report	The report offers recommendation for the management of hearing loss in care homes.
[67]	Echalier et al.	UK	Joining up: why people with hearing loss or deafness would benefit from an integrated response to long-term conditions	2013	Published institute report	The report provides recommendation for the long-term management of hearing loss with dementia.
[59]	Friedner-Woolman et al	USA	Hearing help in nursing homes	2013	Hearing loss magazine	Advice and recommendations aimed at family members seeking to ensure that loved-ones hearing is managed well in care home.
[65]	Flynn et al.	New Zealand	Hearing and vision loss within residential care facilities - the need for improved service delivery	2002	Journal article—report on hearing screening	The study confirms the high prevalence of hearing impairment in long term care and makes a case for increased rehabilitation services being directed towards this population.
[57]	Haque et al.	USA	“There’s a monster under my bed”: hearing aids and dementia in long-term care settings	2012	Web-based article—case management review	Case management of resident with dementia and hearing loss, hearing aid maintenance.
[72]	Hayne et al.	Australia	Acoustic design guidelines for dementia care facilities	2014	Published report for building design	The report gives recommendations for the design of long-term care facilities for people with dementia.
[41]	Hill et al.	UK	What works: hearing loss and healthy ageing	2017	National Health Service England—report	The report gives recommendations based on current research—offering case studies followed by key point recommendation for practice initiatives in hospitals and care homes.
[53]	Hopper et al.	Canada	The relationship between minimum data set ratings and scores on measures of communication and hearing among nursing home residents with dementia	2001	Journal article—quantitative results	Identify residents with minimum data set identified deficits who received a referral for further evaluation.
[27]	Hopper et al.	Canada	Hearing loss among individuals with dementia: barriers and facilitators to care	2012	Journal article—review paper	The review offers interventions to manage hearing loss and dementia which involve staff training, audiology involvement in care homes.
[58]	Hopper et al.	Canada	Hearing loss and cognitive-communication test performance of long-term care residents with dementia: effects of amplification	2016	Journal article—quasi-experimental study	Testing cognitive communication tasks with residents with dementia hearing loss and amplification effects.
[45]	Janes et al.	Canada	Figuring it out in the moment: a theory of unregulated care providers’ knowledge utilization in dementia care settings	2008	Journal article—qualitative study of care staff	The study explores the process whereby staff develop knowledge and utilise person-centred care with people with dementia.
[10]	Jupiter	USA	Cognition and screening for hearing loss in nursing home residents	2012	Journal article—comparison of hearing screening protocols	Determining the accuracy of hearing identified by long-term care staff.
[63]	Jupiter et al.	USA	Perception of hearing loss and hearing handicap on hearing aid use by nursing home residents	1997	Journal article—audiology evaluation	The study examines care home residents’ perception of hearing loss and self-assessed hearing handicap on hearing aid use.
[38]	Lubinski	USA	State of the arts: perspectives on communication in nursing homes	1995	Journal article—review	The review highlights training for staff to raise awareness of hearing-related communication needs of residents.
[68]	Lewsen et al.	Canada	Hearing aids and assistive listening devices in long term care	1997	Journal article—survey of long term care home residents	Study results show that amplification can be used successfully by long-term care residents, as a result of the on-site audiological support.
[55]	McGilton et al.	Canada	Hearing and vision screening tools for long-term care residents with dementia: protocol for a scoping review	2016	Journal article—protocol for pilot study	The pilot study looking at effective screening tool for residents with dementia and communication impairment: hearing and vision.
[44]	McGilton et al.	Canada	Can we help care providers communicate more effectively with persons having dementia living in long-term care homes?	2017	Journal article—mixed methods study	Determine the effects of a communication intervention on residents’ quality of life (QOL) and care, as well as care providers’ perceived knowledge, mood, and burden.
[70]	McManus et al.	UK	Hearing, sound and the acoustic environment for people with dementia	2010	Published institution report	Review: hearing, sound and the acoustic environment for people with dementia.
[49]	Pichora-Fuller et al.	Canada	Helping older people with cognitive decline communicate: hearing aids as part of a broader rehabilitation approach	2013	Journal article—literature review and recommendations	The report calls for a comprehensive planning, including the family and staff teams. This likely to have more success that simply offering hearing aids.
[73]	Pichora-Fuller et al.	Canada	Hard-of-hearing residents in a home for the aged	1994	Journal article—programme evaluation of hearing rehabilitation programme	The participation of older residents with hearing deficits in activities in a home for the aged.
[54]	Pryce	UK	How can you help older people to hear?	2011	Practice journal—review	Need for carers to respond to the complex needs of individuals; staff should take responsibility for managing the environment for better communication.
[18]	Pryce et al.	UK	‘There’s a hell of a noise’: living with a hearing loss in residential care	2012	Journal article—qualitative study	Describes residents’ social interaction affected by hearing loss. The study recommends staff awareness of residents’ communication needs. Attention paid to lack of audiology services.
[47]	Pryce et al.	UK	Foundations of an intervention package to improve communication in residential care settings: a mixed methods study	2013	Journal article—mixed methods exploratory study	Understanding carer knowledge on the impact of hearing loss on residents communication; development of a communication package.
[74]	Ripich	USA	Servicing sensory impaired elderly in long-term care	1995	Book chapter—review of existing practice	Review of the current state of carer encompassing issues around sensory impairment including hearing loss in residential settings.
[69]	Schow	USA	Success of hearing aid fitting in nursing homes	1982	Journal article—intervention study	Development of a programme for hearing rehabilitation and hearing aid servicing in a nursing home.
[26]	Slaughter et al.	Canada	Identification of hearing loss among residents with dementia: perceptions of health care aides	2014	Journal article—qualitative study	Perspectives of care aides (care assistants) on the communication and hearing loss among residents with dementia.
[62]	Solheim et al.	Norway	Lack of ear care knowledge in nursing homes	2016	Journal article—descriptive qualitative study	The objective of the study was to assess the knowledge and skills competence of nursing home staff in relation to the residents’ hearing loss and hearing aids.
[72]	Tolson	UK	Age-related hearing loss: a case for nursing intervention	1997	Journal article—practice review	The review focuses on nursing-led practice in multidisciplinary service developments in the managing age-related hearing loss.
[48]	Weinstein	USA	Hearing loss and senile dementia in the institutionalized elderly	1986	Journal article—hearing assessment intervention	The study sets out to determine the prevalence of hearing impairment in long term care residents with an admitting diagnosis of senile dementia.
[66]	Welsh et al.	Australia	Management of age related hearing loss	2001	Journal article—professional practice review	The review points to best practice in reducing hearing impairment in the elderly.

### Programme theory

Our refined programme theory for achieving optimal hearing-related communication for residents with dementia and hearing loss living in care homes has five components. These are (1) positive regard and empathy for residents—leadership promotion of person-centred care; (2) training on hearing loss and dementia to raise importance of hearing-related communication; (3) knowing the person and awareness; (4) supporting and monitoring of residents’ hearing-related communication needs; and (5) managing noise in the care home environment. Each component within our refined programme theory is presented with its CMOC. Although each CMOC is described independently, they are also inter-related: complexity is the key message.

CMOC 1 (see Table 3) concerns positive regard and empathy for residents. Numerous sources emphasised the importance of understanding the resident as an individual [26, 38–42]. If this is achieved, blanket approaches give way to tailored communication strategies for each individual, such as preferred talking distance, lip reading, and visual cues [38, 43, 44]. It was also evident that good management and leadership are required to achieve effective person-centred care [39, 45] (also suggested by the CEG). Care home leaders need positively to indicate their appreciation towards staff in their efforts to maintaining residents’ personhood (e.g. Beer et al. [39]).

**Table 3:** CMOC 1: Positive regard and empathy for residents—leadership promotion of person-centred care

Programme theory component	Context(s) and interventional strategies suggested by the included papers	Mechanism(s) and outcome(s)	References for data sources
CMOC 1: Positive regard and empathy for residents—leadership promotion of person-centred care	Person-centred care and positive regard to PLWDHL* is seen by care staff to be provided by leadership and management [context]. Care staff are valued [context] and given levels of permission to work autonomously [context]. These contexts are likely to occur when leadership and management model person-centred care and positive regard show they value their staff by enhancing working conditions and providing training opportunities and allow them to work autonomously.	Staff would recognise their professional efficacy and value [mechanism] and feel supported and equipped [mechanism], and believe they have permission to provide person-centred care for PLWDHL [outcome] that is empathetic to their hearing-related communication needs [outcome].	[26, 38–49, 55]

*PLWDHL person living with dementia and hearing loss

We have used the concept of ‘permission’ to capture evidence showing how staff can feel that they have the approval to seek meaningful interaction with residents and to see this as an important part of their work [26, 45, 46]. Leadership and management can intervene in different ways to indicate to staff that they are permitted to practice with a level of autonomy that best serves the hearing-related communication needs of residents: ‘The value of developing close and intimate relationships (with residents) extended into an approach to management of staff which involved reminding staff it’s not all about tasks’ [47] p.32.

Strategies used by leadership that can result in ‘permission’ include appreciating staff needs for relevant training, allowing them to practice with knowledgeable autonomy, and providing resources such as care plans and assessment tools that inform the delivery of person-centred care [44, 48]. Thus, permission results from a context of appropriate leadership ethos and leads staff to recognise their professional competence, so that they engage in effective communication with PLWDHL [45, 49].

CMOC 2 (see Table 4) relates to training around hearing loss, communication, and dementia. A number of papers provided material about communication training for care staff [38, 42–44, 47, 49–52]. Clearly, it is important that staff know about hearing and communication [47]. Important areas to be covered include means of communicating with people who have dementia [26, 27, 49], building communication into a person-centred care approach, and use and maintenance of hearing aids and assistive listening devices [25, 46].

**Table 4:** CMOC 2: Training on hearing loss and dementia to raise importance of hearing-related communication

Programme theory component	Context(s) and interventional strategies suggested by the included papers	Mechanism(s) and outcome(s)	References for data sources
CMOC 2: Training on hearing loss and dementia to raise importance of hearing-related communication	Care staff appreciate the value of [context] and have the skills to address the communication needs of PLWDHL [context]. Strategies may include delivering experiential training that addresses unconscious biases towards ageing, hearing loss, and dementia may be used.	Then, this will enable staff to (1) acknowledge the importance of [mechanism], (2) and feel able to optimise [mechanism] PLWDHL’s hearing communication needs. This is likely to (1) reduce staff feelings of futility [outcomes], (2) reduce PLWDHL’s isolation [outcomes], and (3) increase skills sharing with PLWDHL’s communication partners [outcome].	[25–27, 38, 39, 42–44, 46, 47, 49–53]

However, emphasised by our CEG as well as in the literature, training alone is insufficient without the right attitudes and behaviours. Good quality training may enhance self-efficacy and job satisfaction [39]. Experiential training appears especially potent [39, 44]. It is important that training also deals with any assumptions that staff may have about the futility of some aspects of care, fuelled by unconscious biases towards ageing, dementia, and hearing loss [53]. Similarly, staff may know enough but not do something as it does not seem worthwhile [47].

As with CMOC 1, care home leadership is crucial. Staff need the time to attend training, and also, the culture of the home is important in making them feel that they have time and the authority to practice what they have learned.

CMOC 3 (Table 5) relates to care staff knowledge of the resident as a unique individual. This component depends on having a positive culture in the care home, where relationships between staff and residents (and their families too) are actively encouraged to develop and flourish [26, 51]. Staff will have more knowledge of the resident as an individual, and this will enable them to be considered and bespoke as to how they go about communicating with that individual [26, 42, 51].

**Table 5:** CMOC 3: Knowing the person and awareness

Programme theory component	Context(s) and interventional strategies suggested by the included papers	Mechanism(s) and outcome(s)	References for data sources
CMOC 3: Knowing the person and awareness of hearing communication needs	The care home ethos values relationship-building [context] and staff believe it is legitimate to devote time to relationship building of care staff with PLWDHL and their communication partners [context]. Strategies that are likely to be useful include promoting permissive leadership and awareness-raising training	Staff are more likely to be motivated to invest time in getting to know the PLWDHL [mechanism] resulting in them having personal knowledge of, and rapport with residents [outcome], and will have awareness leading to a person-centred response of challenging behaviours around unmet hearing-related communication needs [outcome].	[18, 26, 27, 39, 40, 42, 44, 47–51, 54–59]

‘Knowing’ the resident means finding out about the things residents hold personally important [27, 54]. This is achieved through spending time caring for people in proximity, learning people’s biography by chatting about their family life or previous career while carrying out care routines [47]. Importantly, ‘knowing the resident’ involves action and responding in ways that promote a person-centred care approach to residents’ hearing-related communication needs [44, 49]. For care staff, there are important experiences of face-to-face understanding, arising from continuous engagement with residents in their daily activities, or through conversations with family carers [39, 40, 42, 49, 51, 55–59]. This demonstration of curiosity to understand the individual resident was more likely to occur in care homes where staff feel they have permission (CMOC 1) to spend time with PLWDHL in meaningful interaction as a core component of their care work [18, 48, 50].

CMOC 4 (Table 6) describes practical measures for supporting residents’ hearing-related communication needs. Several papers in the review described situations where hearing aid use among residents with dementia is often compromised. Little effort is made to ensure that aids are well maintained and working, nor is a priority placed on ensuring residents use their hearing aids [25, 27, 44, 46, 53, 55–58, 60].

**Table 6:** CMOC 4: Supporting and monitoring residents’ hearing-related communication needs

Programme theory component	Context(s) and interventional strategies suggested by the included papers	Mechanism(s) and outcome(s)	References for data sources
CMOC 4: Supporting and monitoring residents’ hearing-related communication needs	Partnerships exist with local hearing services [context]. Workable and resourced procedures exist around monitoring the hearing-related communication needs of PLWDHL [context]. Staff are trained and supported in taking on responsibility for hearing aid use and maintenance [context]. These contexts are likely to develop when leadership, management and care staff, and audiologists get to meet regularly and focus on training and care planning to address the needs of PLWDHL.	Staff are likely to feel confident [mechanism], motivated (buy into) [mechanism], and express self-efficacy [mechanism] to promote residents’ hearing-related communication needs [outcome], provide advocacy, through monitoring residents’ hearing [outcome], and make appropriate referrals to hearing services concerning PLWDHL’s hearing-related communication [outcome].	[25, 27, 38, 41, 44, 46, 49, 50, 53–69]

The numerous reasons for hearing aid non-use include earwax clogging tubes and ear moulds [46, 50, 61–64], depleted batteries [27, 41, 46, 47, 49, 54, 57, 59–63, 65], ill fitting of hearing aids [46, 61, 63], and breakage and loss [27, 38, 46, 49, 56, 60]. Human factors include the time and effort required from staff, with the right know-how, for residents with dementia to become comfortable with fitting and wearing hearing aids [61, 66]. These issues are compounded when staff feel ill-equipped, in terms of training and management support, to deal with hearing aid problems [25, 46].

Appropriate use of hearing aids for PLWDHL may be improved by (1) staff training on the maintenance of hearing aids, as well as effective hearing-related communication training (see CMOC 2) [27, 64, 67], (2) building partnerships between care homes and hearing services (audiology) [27, 58, 68, 69], and (3) care plans that feature the maintenance of hearing aids and support for residents who wear them, with clarity on where responsibility for lies, for example, with keyworker, qualified staff, or communication partners (family) [46, 62, 69]. When these procedures are put in place, they create contexts where important mechanisms are triggered—namely staff are more likely to feel confident in their knowledge of hearing aid use and maintenance [27, 62]—raising their motivation and feelings of self-efficacy to take on responsibility for monitoring hearing aids and ensuring they are working and that residents are wearing them [53, 55, 58, 61]. In addition, with confidence matched with motivation, staff feel able to provide advocacy, through monitoring residents’ hearing [outcome], and make appropriate referrals to services that support the hearing-related communication needs of PLWDHL, such as primary care and audiology services [58].

This CMOC links to CMOC 3; in order to engage fully with residents’ hearing needs, staff need to know the individual and be aware when the resident might need hearing assistive technology in the form of hearing aids or other devices (e.g. wireless headphones for listening to the TV).

CMOC 5 (Table 7) concerns the sound environment of the care home. Managing extraneous noise in the care home environment is crucial to effective resident and staff communication and also that between residents. Ten included papers raised the problem of the noisy environment [18, 29, 38, 47, 54, 58, 61, 65, 66, 68, 70–73]. The impact of background noise on the communication efforts of PWHLD is exacerbated, causing ‘effortful listening’ that diverts cognitive resources from language and memory processing (Hopper et al. [58]). The main culprits are TV and radios; the constant use of which often results from staff believing they are enabling residents’ choice or are simply part of the daily routine [12, 47, 54, 61, 70, 72, 74]. However, kitchen and dining spaces may also be problematic, with ‘poor acoustic conditions for listening (linoleum floors and hard surface) and poor seating for conversation’ [65] (p.143)*.*

**Table 7:** CMOC 5: Managing noise in the care home environment

Programme theory component	Context(s) and interventional strategies suggested by the included papers	Mechanism(s) and outcome(s)	References for data sources
CMOC 5: Managing noise in the care home environment	Staff are knowledgeable of the effects of environmental noise on hearing-related communication [context], recognise the part their routine practice has in reducing noise levels [context], and have permission with time and resources to act [context]. Training (as outlined in CMOC 2), particularly with a focus on the impact of the care home environment, is likely to create the necessary knowledge and recognition of the importance of the care home environment. Permissions to act autonomously may be fostered as explained in CMOC 1.	Staff feel confident to influence the situation [mechanism] and change the physical environment [outcome] (for example) by making changes to living spaces so that they are conducive to hearing-related communication (e.g. chair placement).	[12, 18, 29, 38, 47, 54, 57, 58, 61, 65, 66, 68, 70–74]

When PLWDHL battle against background noise, their listening difficulties can be misconstrued by care staff as problems with cognitive understanding [29, 57]. Careful consideration of the layout and contents of the care home environment and its impact on residents’ listening may warrant a noise audit and steps to reduce it [54, 72]. Furthermore, reducing noise and setting up spaces that show due consideration to the communication needs of PLWDHL may enable the hearing-related communication needs of PLWDHL to be met.

The key mechanism for improving matters is that staff feel confident to take matters in hand, in terms of reducing TV volume or changing the seating arrangements. This is again a question of leadership culture, appropriate training aimed at values and behaviours, and a sense of permission to act.

## Discussion

### Summary of findings

This realist review has developed a programme theory with five sub-components around the optimisation of hearing-related communication for care home residents with dementia and hearing loss. These are positive regard and empathy, communication training, knowing the person, supporting hearing-related communication needs, and managing noise in the environment. Each of these is formulated as a CMOC, developed and refined from the literature and context expert feedback, to reflect what mechanisms act, and when, to produce desired outcomes.

These data generate new insights as to how existing practice may be improved or consolidated and suggests avenues for further research in this area.

**Fig. 2:**
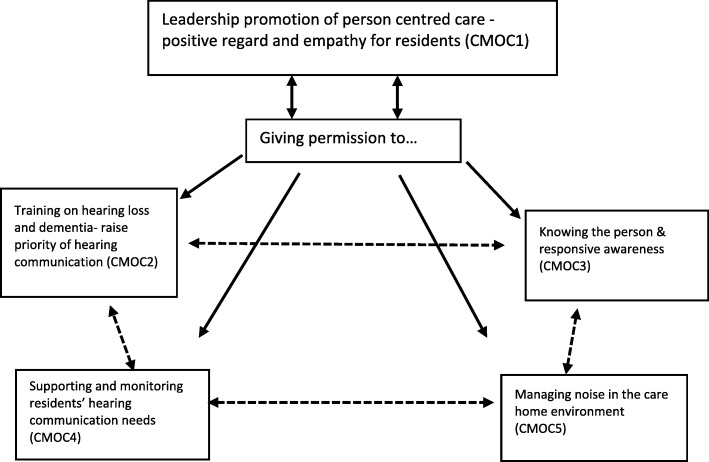
Interaction of permission with five context-mechanism-outcome configurations. The figure illustrates the central position of permission surrounded by the five context-mechanism-outcome configurations (CMOCs 1–5). The solid arrows are the relationship of permission to each CMOC. The broken lines represent the interactions between individual CMOCs. Note that most of these interactions are bi-directional

Perhaps, our most significant result may be found in the way in which these five CMOCs are linked and interacting. There is a thread that runs through much of this paper, which we have conceptualised as ‘permission’. Permission includes notions of having the authority to do something, being empowered, and of making active choices for actions to follow. Permission encapsulates the idea that what care staff consider to be positive actions (the ‘right things to do’) are achieved when they receive permission to undertake the steps needed to provide good care. In the specific area of this review, we have considered permission in relation to hearing-related communication (see Fig. 2), but the mechanism that underpins our concept of permission may be present and ‘triggerable’ in many areas of long-term care. This notion is discussed further in the ‘Comparison with existing literature’ section.

### Strengths, limitations, and future research directions

This review was completed following RAMESES standards for realist reviews [35]. Using this framework, we developed a programme theory in line with available evidence along with reliable contextual expertise from our CEGs. A strength of the study is that through systemic analysis, it provides indications that well-designed dementia communication and hearing aid maintenance training for staff, alongside permissive leadership, could improve care home practices towards optimising hearing-related communication for PLWDHL. A further strength lies in the piloting and subsequent use of a multi-step search strategy. These steps allowed us to refine, refute, and confirm our initial programme theory in the form of five CMOCs.

As a limitation of our study, we recognise that there are multiple contexts, mechanisms, and outcomes embedded within each CMOC. We appreciate that, where possible, when undertaking a realist synthesis, clearer links are made to tie single context features to particular mechanisms which result in defined outcomes. However, this has been difficult to do in our study, partly due to the limited amount and disparate nature of the literature from which we developed and refined our CMOCs. Furthermore, because the evidence for each CMOCs came from a combination of papers, we can at best only tentatively indicate the linkage of our configurations. With only 43 papers in the review, there was limited evidence to support the proposed causal connections, beyond our analytical claims that certain contexts are related to one or more mechanisms and caused certain outcomes. Nevertheless, we were able to partially compensate for the limited relevant data we were able to find. Our CEGs recognised the veracity of our claims within each of the CMOCs and indeed assisted in further refining the potential causal linkage within each. However, it is acknowledged that other experts working with a similar set of data may generate findings that are different.

These lines of argument underpinning the robustness of our CMOCs relies on the agreement between our findings and the world experiences of our experts, as well as the relatively consistent occurrence of events in the papers. However, we acknowledge that our configurations are only demi-regularities [75]. This is to say, they only provide explanations for outcome patterns that are semi-predictable. For example in CMOC 3, training would in some circumstances not result in staff raising their game regardless of better awareness of hearing-related communication deficits among residents. Where there were grounds to suggest that the context and content of training delivery might be impacted by specific interventional strategies, e.g. experiential learning strategies, facilitation by experts, we have attempted to provide some detail of these within our descriptions and explanations of the CMOCs, where the data were available.

We envisage future research would further develop and refine, refute, or confirm aspects of our programme theory and the CMOCs. We believe that a case study approach of selected care homes would allow for closer scrutiny of the CMOCs components, in action; most likely, candidates for workplace development would be staff training and management style. This research would also involve implementation work to normalise relevant CMOC components into routine care.

### Comparison with existing literature

We believe that our concept of permission has a general message for all areas of care within care homes and long-term care institutions for older people. We suggest that permission operates as a guiding and transferable concept for those who manage and work in long-term care institutions for older people. A previous realist review, looking at managing faecal incontinence in care homes (Goodman et al. [76]) discussed how permission acts to enable staff, with the right knowledge, to provide a more person-centred style of care. These authors suggested that care staff training can be a ‘resource for change’, but this can only be effective where staff feel they have permission from the leadership to act upon acquired knowledge and skills. It is also important to keep in mind that hearing-related communication is a subset of the larger issue of communication with people who live with dementia, which in turn is but part of the whole field of person-centred care. Nonetheless, permission for care staff to focus on the individual resident will be important at all these levels.

Our realist synthesis (CMOC 4) also highlights the potential for raising staff confidence and motivation, by care homes creating closer partnerships with audiology services. This is consistent with the findings of Goodman et al. [77], who advocate for stronger partnerships between NHS services and care homes. Where this works well, staff felt they had permission to seek advice on health problems experienced by residents. Having permission taps into staff feelings of autonomy and empowerment to practice in the interests of residents. A review by Squires et al. [78], focusing on job satisfaction of long-term care staff, noted that notions of autonomy and empowerment lent themselves to job satisfaction among staff.

These other studies, however, mention permission only in passing. We wish to be bolder in our assertion that permission—permeated through a context of positive leadership and expressed in care staff feeling emboldened to work in a person-centred way—is the catalyst for positive care practices centred on the hearing-related communication needs of PLWDHL in care home environments. This notion of permissive leadership chimes with McCormack et al. [79], looking at person-centred care in general. The focus of that realist study was on the contextual features of shared decision-making, supportive leadership, and sharing of power to enact innovation and share risks, care staff can derive a sense of permission to own the care delivered to residents with complex needs. These strategies, which echo elements of our programme theory, provide a further analogy to our assertion of the importance of the concept of permission we are advocating. In addition, we believe this concept is potentially transferable, beyond hearing-related communication, to other complex care activities within the care home setting. Therefore, our findings are of interest to anyone concerned with the quality of care in long-term settings and not solely confined to hearing-related communication. However, we appreciate that we have only illustrated the importance of this concept using the specific example of PLWDHL. Whilst this concept may be transferable to other settings and/or conditions, different contextual influences are likely to be at play, potentially resulting in differing outcome patterns. What we do advocate is that this concept may in the future provide a useful lens for future research that seeks to understand practice in care homes and other long term care settings—and possibly beyond.

## Conclusions and recommendations

This realist review has identified five CMOCs that underpin effective hearing-related communication for care home residents with dementia. These findings can be used either to promote good care practice or as testable hypotheses for future research. For example, the CMOCs suggest that emphasis should be placed on leadership development in the care sector; provision of quality training in communication, dementia, and hearing loss; and controlling environmental noise in care homes. For example, this should include studies of practical implementation of the five CMOCs.

In addition, the five CMOCs are linked by the common concept of permission, which we propose is likely to be applicable to any area of long-term care practice, not just hearing and communication. We suggest that permission for staff to do ‘the right thing’, whatever that may be, is fundamental to achieving genuinely person-centred care. This proposition can of course be tested by research looking at other care topics.

## Additional file

Additional file 1: Developing the CMOCs: a worked example. (DOCX 28 kb)

## Abbreviations

CEG: Context expert group
CMOC: Context-mechanism-outcome configuration
PLWDHL: Person living with dementia and hearing loss
RS: Realist evaluation
