# Supplementary material for: Giving permission to care for people with dementia in residential homes: learning from a realist synthesis of hearing-related communication
Source: BMC Med. 2019 Mar 4;17:54. doi: 10.1186/s12916-019-1286-9 (PMC6398258; doi:10.1186/s12916-019-1286-9)
Supplement: Supplementary file 1 — Developing the CMOCs: a worked example. (DOCX 28 kb) [file 12916_2019_1286_MOESM1_ESM.docx]

**Developing the CMOCs: a worked example**

This example of how we developed CMOC 2 (see results) demonstrates the analytical strategy that informed the refinement of all the other CMOCs, and provides text samples from the papers to illustrate the analytical construction of CMO 2.

The CMOC for the staff training component of our initial programme theory stated, in general terms, that providing training on dementia and hearing loss would improve staff understanding and approach to managing hearing-related communication and thus improve communication for individual residents. Some papers included in the review did indeed refer to this. However, even when they did so, many individual papers did not provide detailed accounts of the training delivery, nor was there any discussion of how training might bring about desired outcomes (i.e. the mechanism by which it ‘worked’). Therefore, we looked to build and then refine this CMOC by looking more broadly across all papers for evidence that we could interpret as being relevant data on training that might throw light on aspects of context, mechanisms and outcomes of staff training. From the evidence in the papers we hypothesise that:

**Workshop-based, expert-led, experiential training programmes are reasonable intervention strategies to try as they help care staff to…**

| **Contexts**   1. Appreciate the value of communication needs of people living with dementia and hearing loss (PLWDHL) [context], 2) and have the skills to address these [context].These contexts enable staff to…   **Mechanisms**  1) Acknowledge the importance of [mechanism], 2) and feel able to optimise [mechanism] PLWDHL’s hearing-related communication needs. This is likely to…  **Outcomes**  1) Reduce staff feelings of futility [outcomes], 2) reduce PLWDHL’s isolation [outcomes], 3) and increase skills sharing with PLWDHL’s family [outcome]. | **Illustrative sample text**  *“Staff felt that they beneﬁted from the training, appreciated the opportunity to devote time to observing and interacting with individual residents, and gained a greater understanding of residents.” (1)*  *“The training was felt to be informative and educational, and enabled some carers to feel more effective in their role: ‘‘I have become more observant towards clients.”” (1)*  *“Participants appreciated the impact of communication disability on both the provision of care and the residents’ quality of life. Attention to establishing relationships between residents and health care aides intersects with the broader literature on person-centred care.” (2)*  *“There are potential beneﬁts to patients as well as to NAs [nursing assistants]: the link between person-centered training and increases in general job satisfaction, retention, and a perception of competence has been made.” (3)*  *“[With]* *positive changes in communication behaviour, skills and knowledge… healthcare professionals…were found to use more positive statements… and were rated as being more involved, warmer, less patronising and less likely to show disapproval.” (4)*  *“[Training on communication care planning] can also have beneficial effects on residents’ QOL. The findings evidence that individualized resident-centered interventions improve resident and care provider outcomes.” (5)*  *“Finally, communication training programs have also been shown to improve how family members, caregivers, and nursing aides interact with clients with dementia.” (6)* |
| --- | --- |

**References (from review papers)**

1. Clare L, Whitaker R, Woods RT, Quinn C, Jelley H, Hoare Z, et al. AwareCare: a pilot randomized controlled trial of an awareness-based staff training intervention to improve quality of life for residents with severe dementia in long-term care settings. International Psychogeriatrics. 2013;25(1):128-39. PubMed PMID: 108088306. Language: English. Entry Date: 20130531. Revision Date: 20170928. Publication Type: journal article.

2. Slaughter SE, Hopper T, Ickert C, Erin DF. Identification of hearing loss among residents with dementia: perceptions of health care aides. Geriatr Nurs. 2014 Nov-Dec;35(6):434-40. PubMed PMID: 25212262. English.

3. Beer LE, Hutchinson SR, Skala-Cordes KK. Communicating with patients who have advanced dementia: training nurse aide students. Gerontology & Geriatrics Education. 2012;33(4):402-20. PubMed PMID: 23095223. English.

4. de Vries K. Communicating with older people with dementia. Nursing Older People. 2013;25(4):30-7.

5. McGilton KS, Rochon E, Sidani S, Shaw A, Ben-David BM, Saragosa M, et al. Can We Help Care Providers Communicate More Effectively with Persons Having Dementia Living in Long-Term Care Homes? American Journal of Alzheimer's Disease and other Dementias. 2017;32(1):41-50. English.

6. Pichora-Fuller MK, Dupuis K, Reed M, Lemke U. Helping older people with cognitive decline communicate: Hearing aids as part of a broader rehabilitation approach. Seminars in Hearing. 2013;34(4):308-30. PubMed PMID: 370072732. English.
